# Recoverable and Sensitive Pressure-Induced Mechanochromic Photoluminescence of a Au-P Complex

**DOI:** 10.3390/molecules30092011

**Published:** 2025-04-30

**Authors:** Ningwen Yang, Yijia Chang, Jiangyue Wang, David James Young, Hong-Xi Li, Yuxin Lu, Zhi-Gang Ren

**Affiliations:** 1 College of Chemistry, Chemical Engineering and Materials Science, Soochow University, Suzhou 215123, China; 2 James Watt School of Engineering, University of Glasgow, University Avenue, Glasgow G12 8QQ, UK

**Keywords:** Ag-P complex, stimuli-responsive material, mechanochromic photoluminescence, photoluminescent material

## Abstract

A binuclear Au-P complex [Au_2_(2-bdppmapy)_2_](PF_6_)_2_ (**1**) was synthesised by the reaction of 2-bdppmapy (N,N′-bis-(diphenylphosphanylmethyl-2-aminopyridine) with AuCN and [Cu(MeCN)_4_]PF_6_. The solid phase of **1** emitted bright yellow phosphorescence at *λ*_max_ = 580 nm under UV excitation (QY = 4.41%, τ = 1.88 μs), which shifted to green (*λ*_max_ = 551 nm, QY = 5.73%) after being pressurised under 5 MPa. This colour change was recoverable upon exposure to CH_2_Cl_2_ vapor. Similar mechanochromic photoluminescence behaviour was observed after grinding the crystals of **1**. A filter paper impregnated with **1** demonstrated recyclable write/erase functionality for encrypted information transfer.

## 1. Introduction

Stimuli-responsive complexes with tuneable photoluminescence (PL) have gained considerable attention in recent years [1,2,3,4,5] due to their potential applications as sensors [6,7,8], PL switches [9,10,11], and data storage devices [12,13,14]. Upon exposure to external stimuli, these complexes exhibited visible colour changes in emission, which can be induced by light [15,16,17], electrical current [18,19,20], heat [21,22,23,24], solvent [25,26,27], and mechanical force [28,29,30,31,32,33,34,35,36]. Mechanochromic photoluminescence, a phenomenon where mechanical force alters the emission colour [37,38,39], has been attributed to changes in molecular arrangement [29,40,41], conformational flexibility [42,43], or altered intermolecular interactions [44,45,46]. A number of gold, silver, and platinum complexes emit bright PL in the solid state, and their emission energies can be tuned by variations in metal–ligand coordination bonds, metal–metal interactions, and weak interactions such as van der Waals forces and hydrogen bonds [47,48,49,50,51]. Over the past decades, these complexes have emerged as attractive stimuli-responsive mechanochromic PL materials. For example, an anthryl Au(I) isocyanide complex demonstrated a bathochromic shift in emission from the visible to infrared region upon grinding, attributed to enhanced intermolecular aurophilic interactions [52].

The mechanochromic PL of complexes is most commonly induced by grinding. Under static high pressures (GPa-level), some Cu-N and Pt-N complexes exhibit chromic PL, which recovers to original colours when the pressures are released [53,54,55]. Determining the precise origin of mechanochromic PL is challenging due to the loss of crystallinity complicating solid-state structural characterisation, even though Ito and coworkers reported novel mechano-force-triggered luminescence in Au complexes arising from single-crystal-to-single-crystal domino transformations, while a minor mechano-stimulus is required to trigger the PL [34,35,56].

Various Au(I) complexes with phosphine ligands have exhibited stimuli-responsive PL toward vapor, ions, solvent, and mechano-forces [57,58,59,60], and this has encouraged us to explore the PL behaviours of Au(I) with some hybrid phosphine ligands containing –PPh_2_ groups and other donors such as –Py, –phen, –Pz, C=S, and –C≡CH groups [61,62,63], as the secondary donor group may supply additional sites for coordination and non-covalent interactions that activating the changing over PL. For example, we recently reported that a Au-P-S complex exhibited switchable PL upon grinding, related to the collapse and restoration of the crystalline phase accompanied by the disruption and reforming of intermolecular hydrogen bonds. However, this PL did not change after being treated under static pressure up to 12 MPa [62]. In the current work, we carried out the reaction of the PNP-type ligand 2-bdppmapy (N,N-bis-(diphenylphosphanylmethyl-2-aminopyridine) with AuCN and [Cu(MeCN)_4_]PF_6_, intended to generate some heterometallic Au/Cu complexes with good PL responses. Unexpectedly, this reaction lead to the formation of a new complex [Au_2_(2-bdppmapy)_2_](PF_6_)_2_ (**1**), which contains only two Au(I) metal centres in the structure. The PL of **1** in the solid state changes from yellow to green induced by static pressures as low as 2.5 MPa and can be recovered by exposure to CH_2_Cl_2_ vapor, making it suitable for encrypted information transfer.

## 2. Results and Discussion

### 2.1. Synthesis and Characterisation

**Scheme 1:**
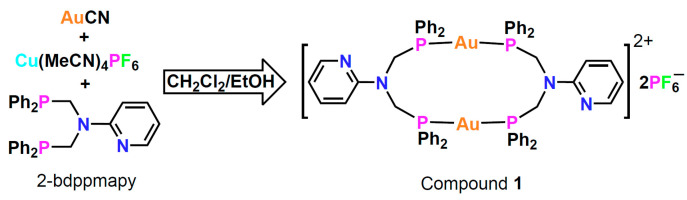
Synthesis of compound **1**.

Crystals of **1**·EtOH were isolated from the reaction of 2-bdppmapy, AuCN, and [Cu(MeCN)_4_]PF_6_ (molar ratio 1:1:1) in CH_2_Cl_2_/EtOH at room temperature followed by the diffusion of petroleum ether and Et_2_O into the reaction mixture (Scheme 1). The driving force for this metathesis was the precipitation of insoluble CuCN. Changing the ratios and salts did not improve the yield.

The EtOH solvent molecules were eliminated quickly from the crystal of **1**·EtOH and resulted in solventless **1** in 58% yield. Compound **1** was stable in air and water, soluble in MeCN, DMF, and DMSO, partially soluble in CH_2_Cl_2_, CHCl_3_, MeOH, and EtOH, and insoluble in other common solvents. Elemental analysis of **1** was consistent with its chemical formula. The TGA curve of **1** (Figure S1) revealed that it had no EtOH solvent molecules at room temperature. It was thermally stable below 250 °C, and subsequently lost its organic components at higher temperatures. The PXRD pattern of **1** generally matched that simulated from the single-crystal X-ray diffraction (SCXRD) data of **1**·EtOH, indicating the main cell parameters remained, whereas the intensity of some peaks varied due to the elimination of EtOH molecules (Figure S2). The IR spectrum of **1** (Figure S3) contained signals attributable to the stretching vibrations of the –Ph and –Py groups at 1591, 1475, 1435, 775, 733, and 691 cm^−1^; of the –CH_2_– group at 1475 cm^−1^; and of the PF_6_^−^ anion at 829 cm^−1^, while that of **1**·EtOH showed the peaks of the lattice EtOH molecule at 3344 cm^−1^ (–OH), 2976, and 2880 cm^−1^ (–C_2_H_5_). The ^1^H NMR spectrum of **1** in DMSO-*d*_6_ (Figure S4) consisted of signals assignable to the –CH_2_– group (4.22 ppm) and the –Py and –Ph groups (8.06–6.57 ppm). The ^31^P{H} NMR spectrum contained a single resonance at 44.66 ppm.

**Figure 1:**
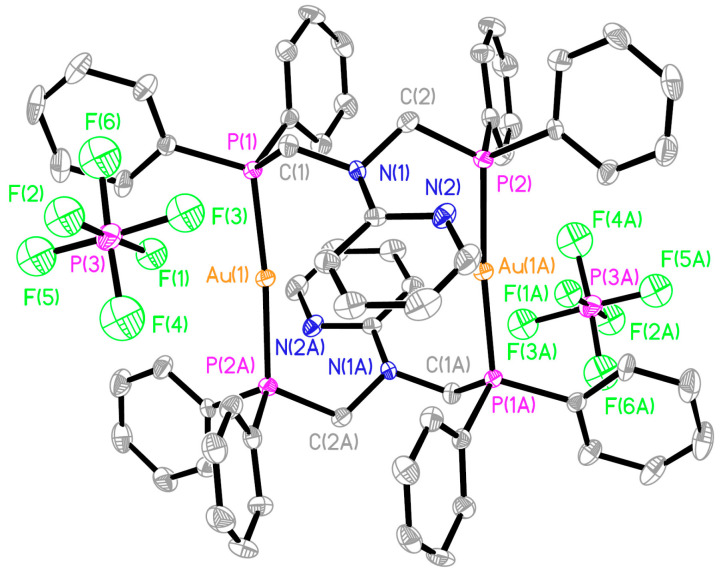
Crystal structure of **1**·EtOH with 30% thermal ellipsoids. All H atoms, disordered F atoms, and the EtOH solvent molecule are omitted for clarity. Symmetry code for A: 1 − x, 1 − y, 1 − z.

SCXRD analysis of **1**·EtOH at 223 K revealed that it crystallised in the space group *P*2_1_/*n*. Each asymmetric unit contains half of the [Au_2_(2-bdppmapy)_2_]^2+^ dication, one PF_6_^−^ anion, and half of the EtOH molecule. Two Au atoms and two 2-bdppmapy molecules are bonded head-to-head to form a distorted Au_2_P_4_ quadrilateral structure (Figure 1). The Au1 atom is coordinated with two –PPh_2_ groups with the P–Au–P bond angle of 174.37(8)°. The mean Au-P bond length is 2.309(2) Å. The distance between Au1 and Au1A (4.550 Å) confirms the absence of an aurophilic interaction.

### 2.2. Photophysical Properties

**Figure 2:**
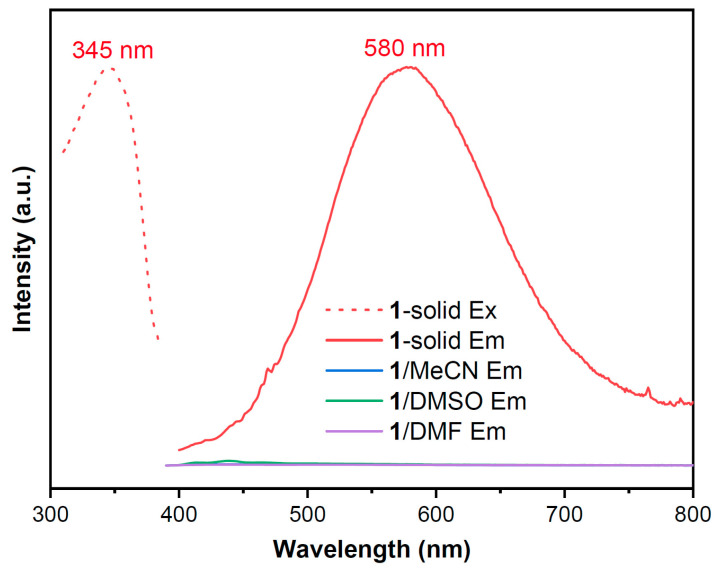
Excitation (dotted line) and emission (solid line) spectra of **1** in the solid state and in solution (1 mg/mL).

The PL responses of **1** in the solid state and in solution at room temperature were recorded (Figure 2). Upon excitation at *λ*_max_ = 345 nm, a solid sample of **1** emitted yellow (*λ*_max_ = 580 nm) with a quantum yield (QY) of 4.41%. The microsecond lifetime (τ = 1.88 μs, excited at 370 nm, measured by transient PL) indicated phosphorescent emission. By comparison, solutions of **1** in MeCN, DMSO, and DMF were non-emissive, likely due to the interactions between **1** and these polar solvents, as well as free rotation of the –Ph groups in solutions that facilitate non-radiative relaxation of the excited state.

**Figure 3:**
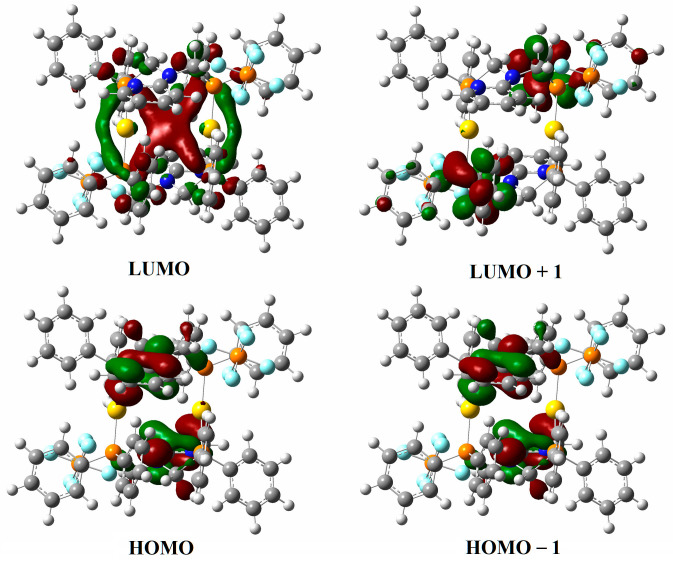
The orbital distributions of HOMOs and LUMOs (isovalue = 0.03) in compound **1**.

Density functional theory (DFT) calculations based on the SCXRD data of **1** were employed to calculate the frontier orbital distributions. As shown in Figure 3, the HOMOs were mainly located at the π orbitals of the –Py group. The LUMO was delocalised over the Au–P bond, while the LUMO+1 was mainly located at the π* orbitals of the –Ph group. The PL of compound **1** is likely due to ligand-to-metal charge transfer (^3^LMCT) combined with intra-ligand charge transfer (^3^ILCT) [64,65].

### 2.3. Pressure-Induced Mechanochromic Photoluminescence

**Figure 4:**
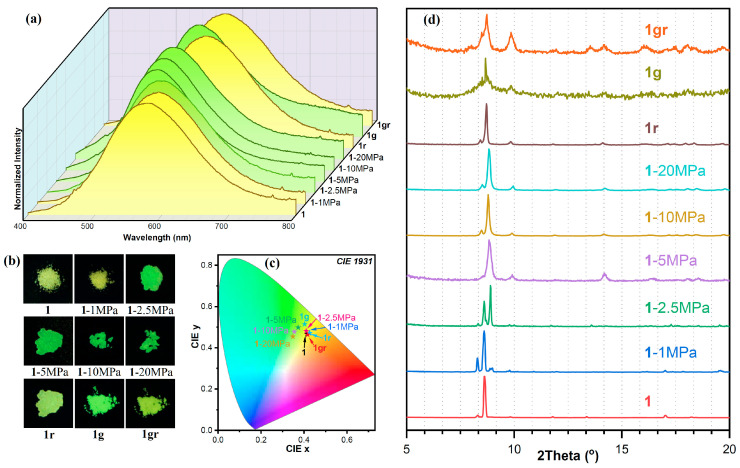
Comparisons of (**a**) emission spectra (excited at 354 nm), (**b**) photos under 365 nm LED irradiation, (**c**) CIE plot, (**d**) PXRD patterns of **1**, in response to different pressures for 10 min (**1**—1 MPa, **1**—2.5 MPa, **1**—5 MPa, **1**—10 MPa, **1**—20 MPa), **1r** (**1**—5 MPa after exposure to CH_2_Cl_2_ vapor), **1g** (**1** ground in a mortar), and **1gr** (**1g** after exposure to CH_2_Cl_2_ vapor).

We investigated the PL of five samples of **1** after exposure to different pressures (**1**—1 MPa, **1**—2.5 MPa, **1**—5 MPa, **1**—10 MPa, **1**—20 MPa) for 10 min (Figure 4a). The emission *λ*_max_ of **1** remained unchanged in response to low pressure (**1**—1 MPa, 580 nm), but shifted to shorter wavelengths in response to higher pressures (**1**—2.5 MPa, *λ*_max_ = 573 nm; **1**—5 MPa, *λ*_max_ = 551 nm; **1**—10 MPa, *λ*_max_ = 547 nm; and **1**—20 MPa, *λ*_max_ = 547 nm). Accompanied with a slightly enhanced QY (5.73% for **1**—5 MPa), this yellow-to-green colour change was visible to the naked eye (Figure 4b,c).

PXRD patterns were obtained to examine the phase transition of **1** in response to pressure. In the PXRD patterns of **1**—5 MPa, **1**—10 MPa, and **1**—20 MPa, the major peaks at 2θ = 8.3° (h, k, l = 0, 1, 1), 8.6° (h, k, l = 1, 0, 1), 9.8° (h, k, l = 1, 1, 0) vanished, while peaks at 8.5°, 8.8° and 9.9° appeared. The changing on PXRD peaks indicated the change in interplanar crystal spacing distances and the forming of a new crystalline phase. By comparison, the PXRD pattern of **1**—2.5 MPa contained peaks corresponding to both phases, demonstrating incomplete phase-transition. In the case of **1**—1 MPa, weak signals at 8.9° and 9.0° were also observable, illustrating that this phase transition could occur slightly even at low pressure.

Since the changes in the emission spectra and PXRD patterns were almost complete after 10 min at 5 MPa, we used this sample as a model. When **1**—5 MPa was exposed to CH_2_Cl_2_ vapor for 5 min, the resulting solid (**1r**) emitted at 582 nm, almost identical to the PL of **1**. Likewise, the PXRD pattern of **1r** showed peaks at 2θ = 8.4°, 8.7°, and 9.8°, again almost identical to **1** (Figure 4a,c). This cycle of pressure and exposure to CH_2_Cl_2_ vapor was repeated five times without noticeable changes in the PL wavelengths of the two phases (Figure S5).

When **1** was ground in a mortar, the emission of the resulted solid **1g** blue-shifted to *λ*_max_ = 557 nm, in-between the wavelengths of **1**—2.5 MPa and **1**—5 MPa (Figure 4a), indicating that grinding had less of a mechanochromic PL effect than pressure at 5 MPa. When **1g** was exposed to CH_2_Cl_2_ vapor, the emission of the resulted solid (**1gr**) also recovered to *λ*_max_ = 580 nm. Although the PXRD patterns of **1g** and **1gr** were less crystalline, some peaks were observable. The PXRD pattern of **1g** showed peaks at 2θ = 8.5°, 8.7°, and 9.9°, which recovered to 2θ = 8.5°, 8.7°, and 9.8° in **1gr**. These emission spectra and PXRD patterns indicated that the phase transition induced by grinding was broadly similar to that induced by static pressure.

**Figure 5:**
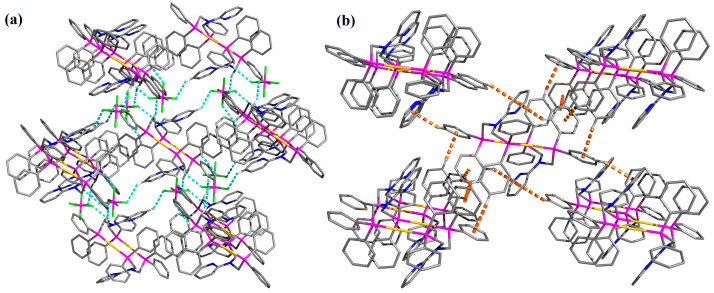
(**a**) Hydrogen bonds (dashed light-blue lines) between the [Au_2_(2-bdppmapy)_2_]^2+^ dication and the PF_6_^−^ anions. All non-hydrogen-bonded H atoms are omitted for clarity; (**b**) C–H···π interactions (dashed yellow lines) between neighbouring [Au_2_(2-bdppmapy)_2_]^2+^ dications. All hydrogen atoms and PF_6_^−^ anions are omitted for clarity. Atom colors: Au, light-yellow; P, magenta; N, blue; C, gray; F, green; H, white.

To explain these phase transitions, we investigated the SCXRD data and calculated that there was a total void of 204.2 Å^3^ in each cell (Figure S6, 6.3% of cell volume, calculated using PLATON v1.18) in the crystal structure of **1**. We then performed nitrogen adsorption/desorption experiments which indicated that the pore volume of **1** (0.042 cm^3^/g) was reduced to nearly half in **1**—5 MPa (0.021 cm^3^/g), and was restored in **1r** (0.047 cm^3^/g). We therefore concluded that pressure reduced the void in the crystal structure in **1**, and CH_2_Cl_2_ molecules then inserted into the voids and restored the unit cell, which has been observed in other mechanochromic Au complexes [66,67,68]. Examination of the SCXRD data also indicated various C–H···F hydrogen bonds (Table 1 and Figure 5a) between the [Au_2_(2-bdppmapy)_2_]^2+^ dication and the PF_6_^−^ anions, and a number of intra- and inter-molecular C–H···π interactions (Table 2 and Figure 5b) between neighbouring [Au_2_(2-bdppmapy)_2_]^2+^ dications.

**Figure 6:**
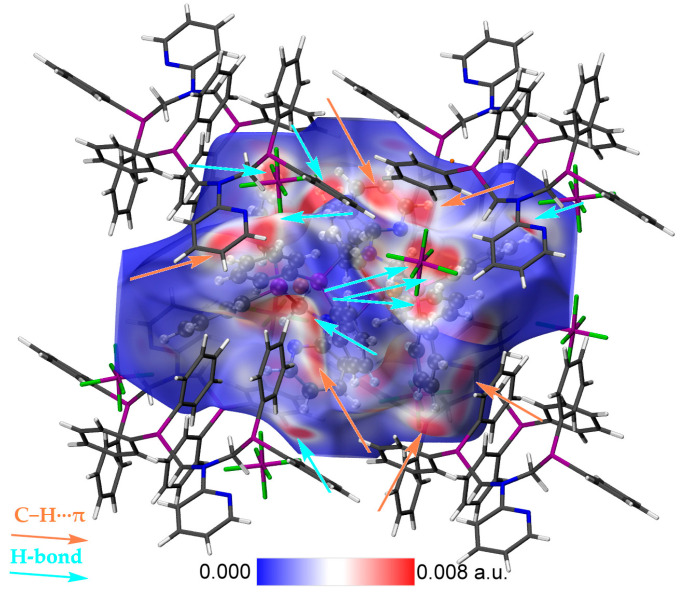
The Hirshfeld surface analysis of compound **1**. Light-blue and yellow arrows directed to the hydrogen bonds and C–H···π interactions, respectively.

We examined the Hirshfeld surfaces to quantify the contribution of non-covalent interactions in the crystal packing. The software Multiwfn v3.8 [69] was used for the analysis of the Hirshfeld surface. As shown in the coloured map of *d*_norm_ (Figure 6), the red-coloured regions signified the presence of the aforementioned hydrogen bonds and C–H···π interactions, which were consistent with the results obtained from PLATON calculations.

**Figure 7:**
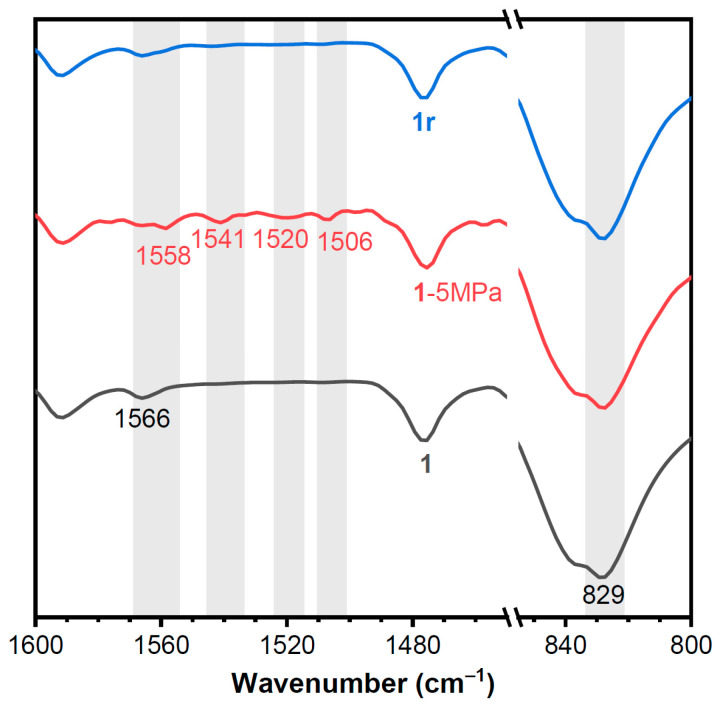
Comparison of the IR spectra of **1**, **1**—5 MPa, and **1r** in the range 800–1600 cm^−1^.

Comparing the IR spectra of **1** and **1**—5 MPa (Figure 7), the characteristic absorption of PF_6_^−^ at 829 cm^−1^ remained unchanged. However, the signals for the –Ph and –Py groups at 1506 cm^−1^ distinctly weakened in the latter, and some new weak signals at 1558, 1541, 1520, and 1506 cm^−1^ appeared. These IR spectral data suggest that during mechanical pressuring, the hydrogen bonding environment of the PF_6_^−^ anion was minimally affected, whereas the chemical environment around the –Ph and –Py groups was significantly affected. These spectral changes were restored in **1r**, consistent with the restoration of the emission spectra. To this end, we propose that pressure mostly disrupts the C–H···π interactions associated with the –PPh_2_ and –Py groups, thereby altering the emission of **1** in the solid state.

### 2.4. Application to Encrypted Information Transfer

**Figure 8:**
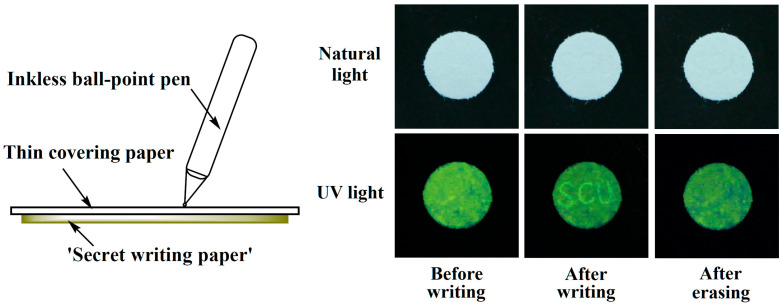
(**left**) Schematic plot of the encrypted information transfer experiment; (**right**) photos of the ‘secret writing paper’ under natural and UV (365 nm) light before/after writing ‘SCU’ and erasing with CH_2_Cl_2_ vapor.

This responsiveness of the PL of complex **1** to external stimuli was used to make invisible ink [70]. Compound **1** was impregnated on filter paper and activated by CH_2_Cl_2_ vapor. Writing on a thin cover paper placed over the filter paper using an inkless ball-point pen (Figure 8) at normal writing strength did not produce a noticeable change under natural light. However, writing appeared under UV light at 365 nm, and could be erased by exposure to a CH_2_Cl_2_ atmosphere for 5 min. This write/erase operation could be repeated several times and endowed compound **1** as an encrypted information transformation material.

## 3. Experimental Section

### 3.1. Materials, Characterisation, and Measurements

2-Bdppmapy was prepared using a method from the literature [71]. All other materials were supplied from commercial sources and used as received. Elemental analyses were performed on a Thermal Fisher Flash Smart microanalyzer (Thermo Fisher Scientific, Waltham, MA, USA). Powder X-ray diffraction (PXRD) patterns were recorded on a Bruker D2 Phaser X-ray diffractometer (Bruker, Billerica, MA, USA) with the Bragg–Brentano method using a Cu *K*α source (30 kV, 10 mA). IR spectra were acquired on a Bruker VERTEX 70 FT-IR spectrometer (4000–600 cm^−1^) (Bruker, Billerica, MA, USA) with an ATR probe. Thermogravimetric analysis (TGA) was performed on a TA SDT-2960 analyser (TA Instruments, New Castle, DE, USA) from room temperature to 800 °C under a N_2_ stream, with a heating rate of 10 °C /min. NMR spectra were acquired on a Bruker AVANCE NEO NMR spectrometer (Bruker, Billerica, MA, USA). N_2_ adsorption/desorption experiments were conducted using a Belsorp-Max gas adsorption analyser (Microtrac BEL, Osaka, Japan). Emission spectra, transient photoluminescence, and QY measurements were performed on an Edinburgh FLS1000 spectrometer (Edinburgh Instruments, Livingston, UK).

### 3.2. Synthesis of [Au_2_(2-Bdppmapy)_2_](PF_6_)_2_ (***1***)

A mixture of CH_2_Cl_2_/EtOH (*v*/*v* = 1:1, 6.0 mL), AuCN (8.92 mg, 0.04 mmol), and 2-bdppmapy (19.6 mg, 0.04 mmol) was stirred at room temperature for 6 h, followed by the addition of [Cu(MeCN)_4_]PF_6_ (14.6 mg, 0.04 mmol) and more stirring for 5 min. The resulting suspension was then filtered, and the filtrate was diffused with petroleum ether and Et_2_O (1:1). [Au_2_(2-bdppmapy)_2_](PF_6_)_2_·EtOH (**1**·EtOH) was isolated as colourless crystals after 2 days, which were collected, washed with Et_2_O, and dried in air. Yield for **1**: 76.7 mg (58% based on Au). Anal. Calcd for C_62_H_56_Au_2_F_12_N_4_P_6_: C, 44.73; H, 3.39; N, 3.37; found: C, 43.93; H, 3.70; N, 3.24 (%). IR (ATR, cm^−1^): 1591 (m), 1566 (w), 1476 (m), 1435 (s), 1283 (w), 1221 (m), 1159 (w), 1099 (m), 829 (vs), 775 (m), 733 (s), 691 (s). ^1^H NMR (400 MHz, DMSO-*d*_6_, ppm): *δ* 8.06 (dd, *J* = 4.9, 1.3 Hz, 2H), 7.44–7.36 (m, 42 H), 6.75 (d, *J* = 8.6 Hz, 2H), 6.57 (dd, *J* = 6.9, 5.0 Hz, 2H), 4.22 (d, *J* = 2.4 Hz, 8H). ^13^C NMR (101.6 MHz, DMSO-*d*_6_, ppm): *δ* 156.62, 147.20, 137.15, 136.97, 132.98, 132.89, 132.79, 128.93, 128.62, 128.59, 128.56, 112.12, 107.40, 49.82 ppm. ^31^P{H} NMR (162 MHz, DMSO-*d*_6_, ppm): *δ* 44.66.

### 3.3. Preparations of ***1***—1 MPa, ***1***—2.5 MPa, ***1***—5 MPa, ***1***—10 MPa, ***1***—20 MPa, and ***1g***

A mould (Φ = 9 mm) containing 3 mg of **1** was subjected to pressures of 1 MPa, 2.5 MPa, 5 MPa, 10 MPa, and 20 MPa (hand press) for 10 min each. The pressure was then released and the pellets collected (**1**—1 MPa was collected as a powder due to the low pressure). Compound **1g** was prepared by grinding **1** (3 mg) in a mortar for more than 5 min.

### 3.4. Preparations of ***1r*** and ***1gr***

Samples of **1**-5 MPa and **1g** were placed in a small beaker (5 mL), and sealed with 5 mL of CH_2_Cl_2_ in a 50 mL beaker for 5 min, respectively.

### 3.5. Preparation of ‘Secret Writing Paper’

Powdered **1** (3 mg) was plastered onto a small filter paper (Φ = 9 mm). This ‘secret writing paper’ was exposed to CH_2_Cl_2_ vapor for 5 min to activate it before use.

### 3.6. Single-Crystal X-Ray Diffraction (SCXRD) Determination

A single crystal of **1**·EtOH (0.30 mm × 0.30 mm × 0.10 mm) was selected directly from the synthesis. SCXRD measurements were performed on an Agilent Xcalibur diffractometer (Agilent, Santa Clara, CA, USA) using Mo *K*α (*λ* = 0.71073 Å) radiation at 223 K. The diffraction data were collected and refined, and a multi-scan absorption correction was applied using CrysAlisPro 1.171.42.81a. The structure was solved by direct methods using SHELXS (Sheldrick, 2016/6) and refined by full-matrix least-squares methods against *F*^2^ using SHELXL (Sheldrick, 2016/6) [72]. The PF_6_^−^ anion was disordered over two sites rotating along the F1-P3-F2 axis with occupancies of 0.50/0.50 for F3-F6/F3A-F6A. The EtOH mole was disordered at opposite positions with equal (0.5/0.5) occupancies. The disordered F atoms at the PF_6_^−^ anion and the C and O atoms of the disordered EtOH molecule were refined isotropically, while all other non-hydrogen atoms were refined anisotropically. Hydrogen atoms were placed at calculated positions and constrained to ride over their parent C and N atoms. Selected crystallographic data and refinement parameters are listed in Table S1.

### 3.7. Computational Details

Theoretical calculations were conducted using the Gaussian 16-C01 software package at the B3LYP-GD3BJ/def2SVP level [73]. Based on the crystal structure, we froze the heavy atoms in the optimisation and optimised the hydrogen atoms.

## 4. Conclusions

In summary, we have synthesised a binuclear Au-P complex [Au_2_(2-bdppmapy)_2_](PF_6_)_2_ (**1**), which emitted yellow phosphorescence in the solid state at *λ*_max_ = 580 nm upon 345 nm excitation. DFT calculations suggested that this PL is attributable to a combination of ^3^LMCT and ^3^ILCT. Induced by pressures as low as 2.5 MPa, the emission of **1** visibly shifted from yellow to green. The PXRD pattern changes indicated a clear phase transition, observable at 1 MPa and completed at pressures exceeding 5 MPa. This emission change could be recovered by exposure to CH_2_Cl_2_ vapor, and these reversible transformations could be cycled multiple times. The mechanochromic PL behaviour of **1** was likely associated with the reducing of voids in the unit cell, which altered the inter- and intra-molecular C–H···π interactions, perturbed electron densities over the –PPh_2_ and –Py groups, and varied energy gaps between the excited and ground states. Furthermore, a ‘secret writing paper’ impregnated with complex **1** was utilised to make invisible ink that can be seen under UV light and erased upon exposure to CH_2_Cl_2_ vapor. This work presented an example of a novel recoverable mechanochromic PL material suitable for force-sensitive sensors. Additionally, our laboratory is actively exploring other stimuli-responsive photoluminescent complexes.

## Figures and Tables

**Table 1:** Hydrogen bond lengths (Å) and angles (°) in **1** (calculated by PLATON v1.18).

D–H···A	D–H	H···A	D···A	D–H···A
C1–H1B···F3	0.98	2.42	3.35(2)	157
C2–H2A···F6 ^i^	0.98	2.48	3.09(2)	120
C6–H6···F4 ^ii^	0.94	2.43	3.21(2)	141
C21–H21···F2 ^i^	0.94	2.51	3.34(2)	147

Symmetry codes: (^i^) 0.5 − x, −0.5 + y, 1.5 − z; (^ii^) −x, 1 − y, 1 − z.

**Table 2:** C–H···π interaction distances (Å) and γ angles (°) in **1** (calculated by PLATON v1.18).

D–H···Cg	H···Cg	γ
C9–H9···Cg1 ^i^	2.76	7.7
C17–H17···Cg1 ^ii^	2.84	10.9
C22–H22···Cg2 ^iii^	2.91	11.9
C23–H23···Cg3 ^iii^	2.86	8.7

Cg1: N2, C3–C7; Cg2: C8–C13; Cg3: C14–C19; Symmetry codes: (^i^) 1 − x, 1 − y, 1 − z; (^ii^) 0.5 − x, 0.5 + y, 1.5 − z; (^iii^) 1.5 − x, −0.5 + y, 1.5 − z.

## Data Availability

The crystallographic data are available from the Cambridge Crystallographic Data Centre (CCDC number 2419373). Other data not presented in the Supplementary Materials are available on request from the corresponding author.

## Supplementary Materials

The following supporting information can be downloaded at https://www.mdpi.com/article/10.3390/molecules30092011/s1, Figure S1: TGA curve of **1**; Figure S2: PXRD patterns of as-synthesised **1** and those simulated from the SCXRD data; Figure S3: IR spectra of **1** and **1**·EtOH; Figure S4: ^1^H, ^13^C, and ^31^P{^1^H} NMR spectra of **1** in DMSO-*d_6_*; Figure S5: Emission spectra and maximum wavelength of the emission spectra of **1** during the 5-round pressure-vapor cycles; Figure S6: Packing diagram indicating the void in **1**; Table S1: Selected crystallographic data and refinement parameters for **1**. Cartesian coordinates of molecule **1**.
