# Supplementary material for: Recoverable and Sensitive Pressure-Induced Mechanochromic Photoluminescence of a Au-P Complex
Source: Molecules. 2025 Apr 30;30(9):2011. doi: 10.3390/molecules30092011 (PMC12073243; doi:10.3390/molecules30092011)
Supplement: Supplementary file 1 [file molecules-30-02011-s001.zip › molecules-3572372-supplementary.pdf]

Contents

Figure S1. TGA curve of **1**.

Figure S2. PXRD patterns of as-synthesized **1** and that simulated from the SCXRD data.

Figure S3: IR spectra of **1** and **1**·EtOH.

Figure S4. ^{1}H, ^{13}C and $^{31}P\{^{1}H\}$ NMR spectra of **1** in DMSO-d_6.

Figure S5. Emission spectra and maximum wavelength of the emission spectra of **1** during the 5-round pressure-vapor cycles.

Figure S6. Packing diagram indicating the void in **1**.

Table S1. Selected crystallographic data and refinement parameters for **1**·EtOH.

Cartesian coordinates of the molecule of **1**

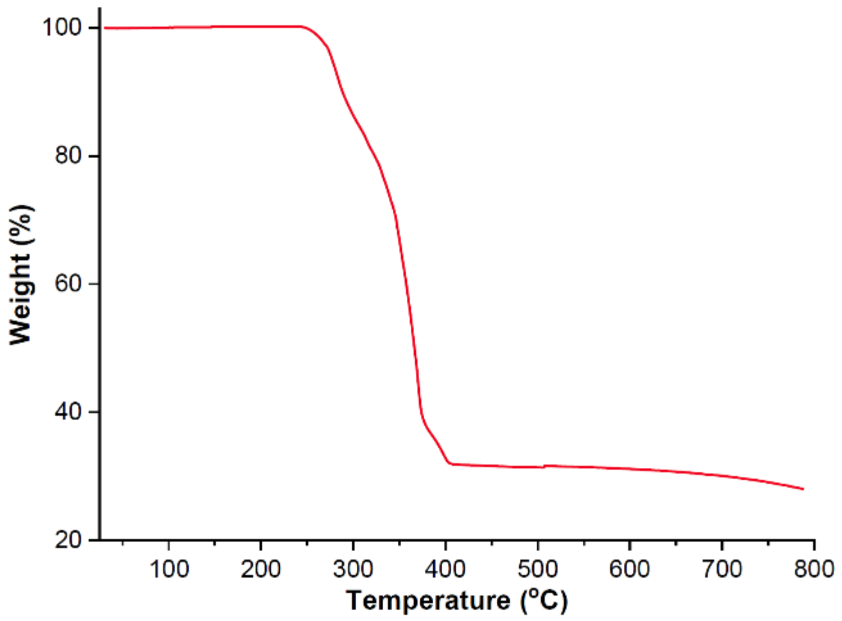

Figure S1. TGA curve of **1** in a N_2 stream.

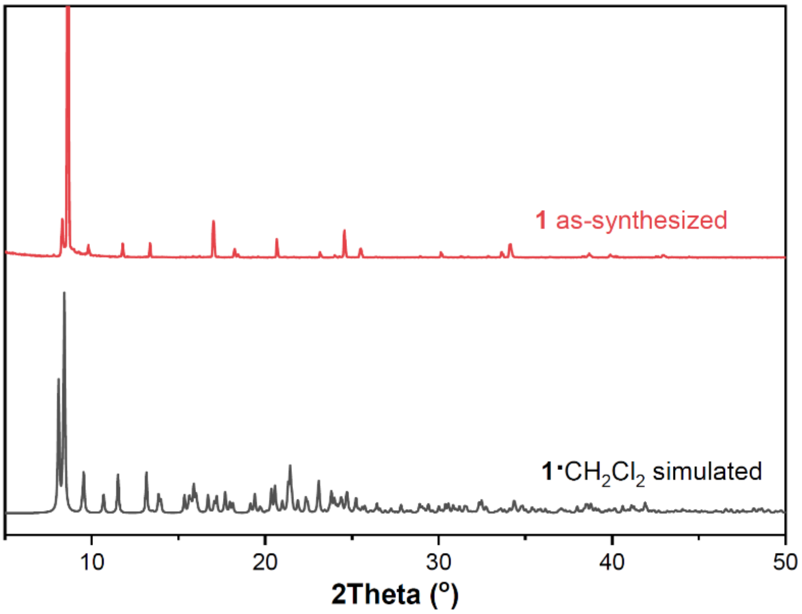

Figure S2. PXRD patterns of as-synthesized **1** (red curve) and that simulated from the SCXRD data of **1**·CH_2Cl_2 (black curve).

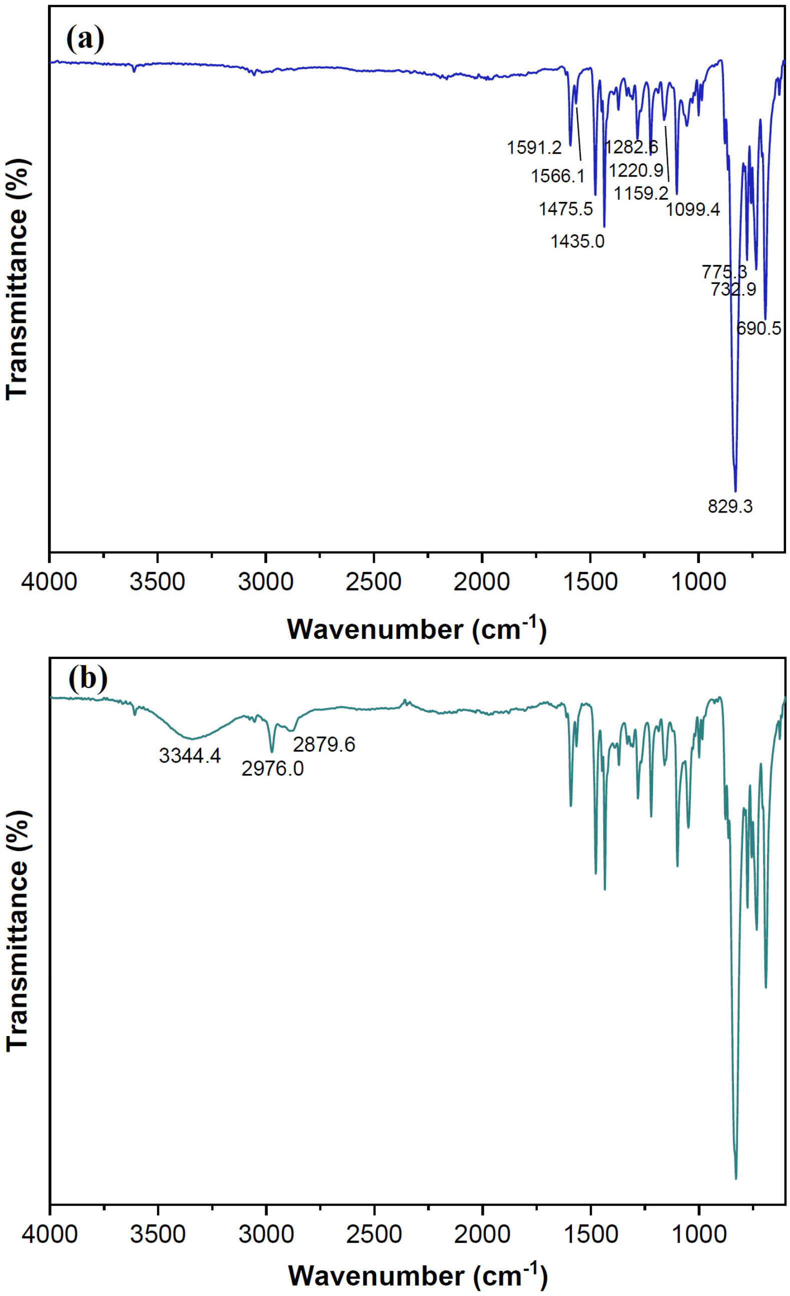

Figure S3. IR spectra of (a) **1** and (b) **1**·EtOH

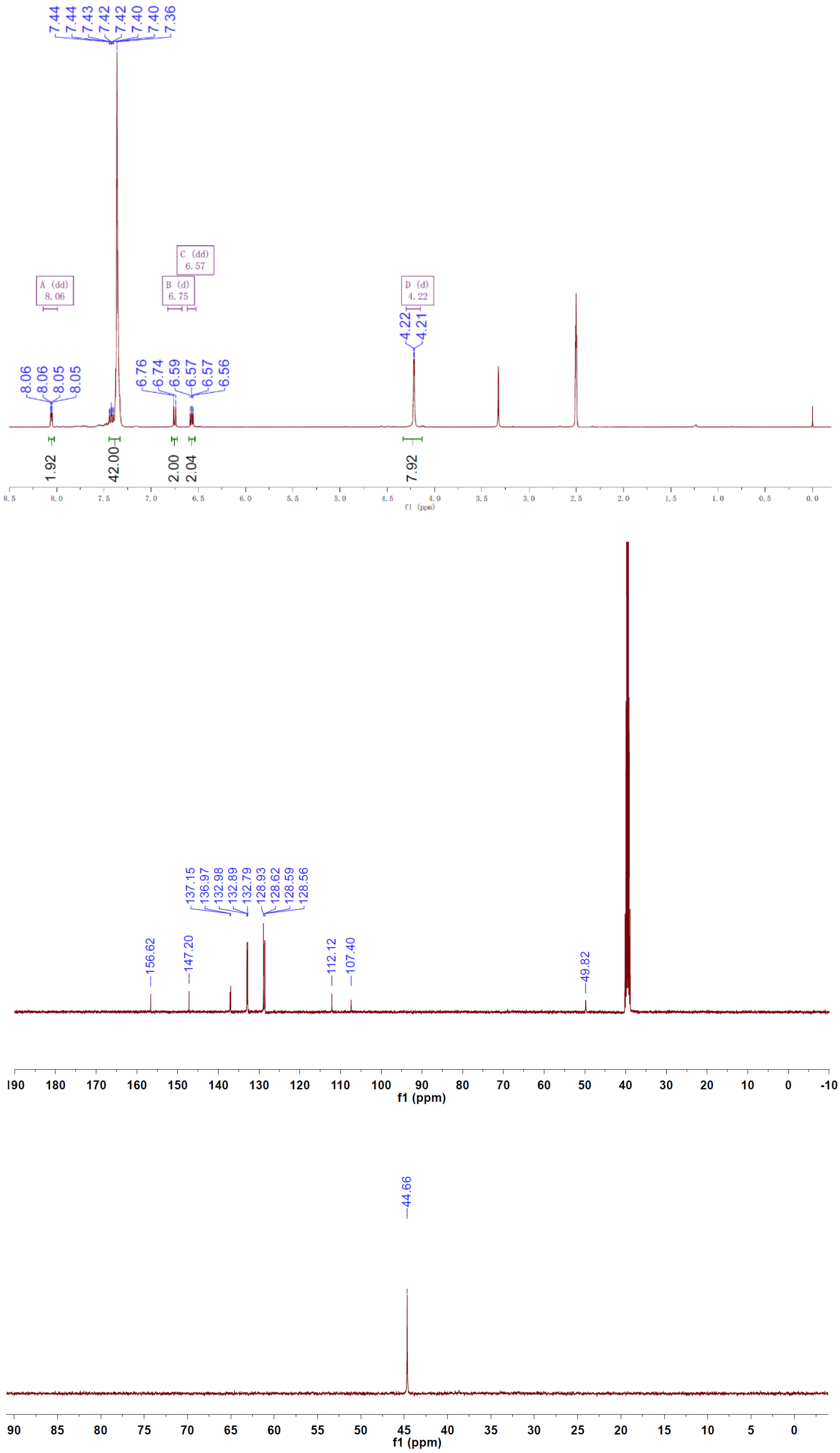

Figure S4. ^{1}H, ^{13}C and $^{31}P\{^{1}H\}$ NMR spectra of **1** in DMSO-d_6.

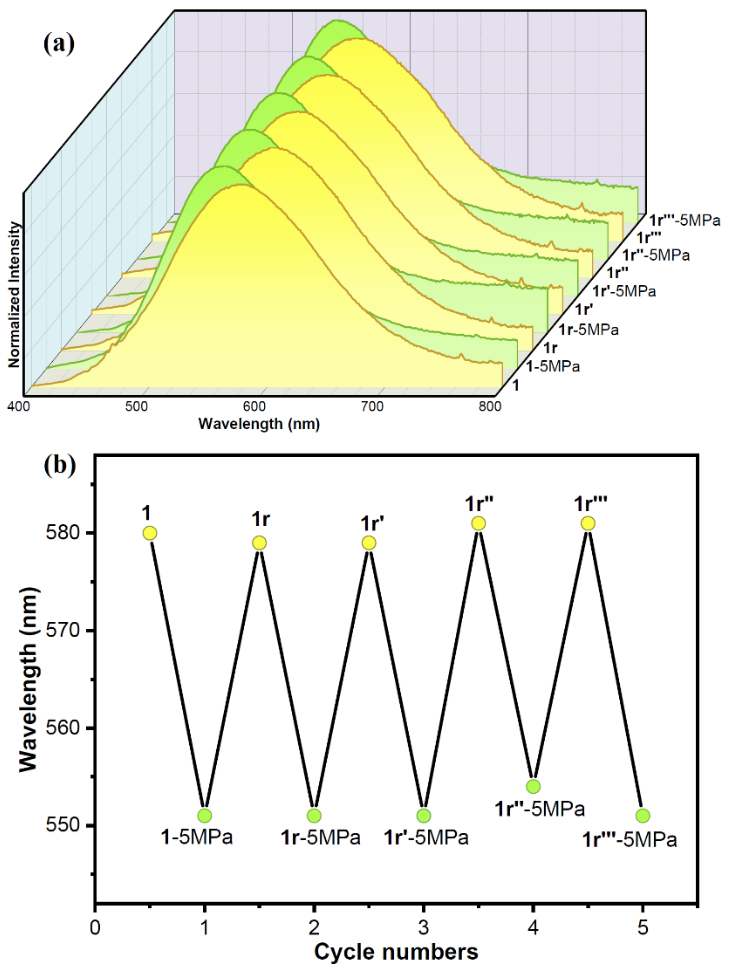

Figure S5. (a) Emission spectra and (b) maxima wavelength of the emission spectra of **1** during the 5-round pressure-vapor cycles.

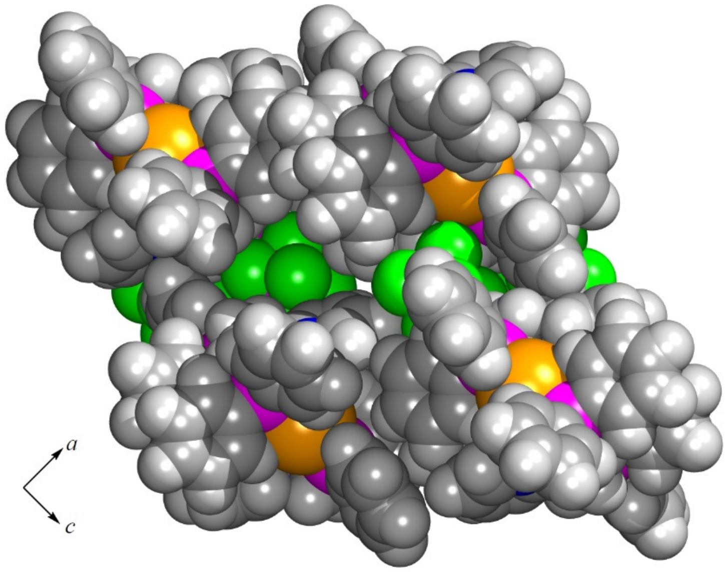

Figure S6. Packing diagram indicating the void in **1**, viewed along the *b* axis.

Table S1. Selected crystallographic data and refinement parameters for **1**·EtOH.

CCDC No.	2419373
Formula	$C_{64}H_{62}Au_2F_{12}N_4OP_6$
Formula weight	1710.93
Temperature/K	223(2)
Wavelength/Å	0.71073
Crystal system	monoclinic
Space group	$P2_1/n$
a/Å	12.7809(7)
b/Å	13.4708(9)
c/Å	18.6970(11)
β/°	90.657(5)
V/Å^3	3218.8(3)
Z	2
ρ_{calc}/g.cm^3	1.765
F(000)	1676
μ/mm^{-1}	4.782
Index ranges	$-16 \leq h \leq 16$, $-16 \leq k \leq 17$, $-23 \leq l \leq 24$
Crystal size/mm^3	0.30 × 0.30 × 0.10
Reflections collected	19213
Independent reflections	7647
R_{int}	0.0545
Data / restraints / parameters	7647 / 2 / 399
R_1[a]	0.0506
wR[b]	0.1212
GOF[c]	1.074
residual peaks/e Å^{-3}	2.579, -0.972

[a] $R_1 = \Sigma||F_o|-|F_c||/\Sigma|F_o|$. [b] $wR_2 = \{\Sigma w(F_o^2-F_c^2)^2/\Sigma w(F_o^2)^2\}^{1/2}$. [c] $GOF = \{\Sigma w((F_o^2-F_c^2)^2)/(n-p)\}^{1/2}$, where n = number of reflection and p = total number of parameters refined.

Cartesian coordinates of the molecule of 1 corresponding to the energy minimum.

Au	5.90123767	8.50556744	10.31499394
P	5.54778476	7.63953556	12.44720368
P	5.87644964	3.38408640	10.47717351
N	4.27011413	5.46659530	11.43606278
N	3.31313697	4.24228929	9.73731381
C	4.12569628	6.45976942	12.48115986
H	4.08027904	5.96260178	13.46800075
H	3.19884961	7.04437658	12.35813523
C	4.91053648	4.22129744	11.82205685
H	4.16698067	3.48667629	12.18004183
H	5.61077114	4.43707518	12.64476375
C	3.38822247	5.43121580	10.36849114
C	2.61522426	6.54229148	9.97397706
H	2.74117456	7.51758268	10.44402800
C	1.63934976	6.34151055	9.00539268

H	0.98690676	7.17546245	8.72739062
C	1.49854716	5.08746397	8.39393893
H	0.73290989	4.89640748	7.63971633
C	2.39391353	4.08942496	8.76587895
H	2.37306325	3.10333709	8.28802784
C	6.95435374	6.69409377	13.09733542
C	8.10472316	6.53383488	12.30853809
H	8.13364193	6.95266820	11.29644388
C	9.20661251	5.84301834	12.81858698
H	10.09695242	5.71032269	12.20096021
C	9.16621743	5.31340347	14.10934740
H	10.03144637	4.76826710	14.49511336
C	8.01704799	5.45612880	14.89157202
H	7.96833222	5.02507178	15.89415353
C	6.91009288	6.13837158	14.38889477
H	6.01395623	6.22085010	15.00902929
C	5.16493818	8.89384559	13.69501262
C	4.56458436	8.54982455	14.91836208
H	4.24454423	7.52481220	15.12609529
C	4.35984887	9.52881680	15.89246122
H	3.88268522	9.26097325	16.83869445
C	4.74707286	10.85156010	15.65275767
H	4.59581779	11.61433758	16.42284427
C	5.30836868	11.20631135	14.42203184
H	5.58194587	12.24720925	14.22636919
C	5.51202349	10.23048763	13.44356864
H	5.92363720	10.50187350	12.46472129
C	7.50854352	3.07428814	11.22015286
C	7.66018923	2.58358903	12.52952790
H	6.79831756	2.45245417	13.18942693
C	8.93392466	2.27776558	13.01178139
H	9.05018293	1.88457843	14.02550691
C	10.05922739	2.46661560	12.20268793
H	11.05330707	2.22545314	12.58598591
C	9.91788479	2.97287522	10.90939953
H	10.79169488	3.11488397	10.26754815
C	8.64498374	3.27821932	10.42207883
H	8.52169650	3.66023021	9.40200833
C	5.07996590	1.76012257	10.33509965
C	4.75383416	1.23385672	9.07671111
H	5.02369665	1.79868317	8.17724891
C	4.07693729	0.01498950	8.98088516
H	3.81911477	-0.39928538	8.00150024
C	3.72211756	-0.67623591	10.14388672
H	3.16753048	-1.61441935	10.08225446
C	4.06819350	-0.16750646	11.39820187
H	3.79029909	-0.72347285	12.29675492
C	4.74706719	1.04749561	11.49853923
H	4.98452027	1.44447941	12.48930926
P	1.31797211	9.60545852	12.29948661
F	2.84493463	9.39784720	11.72644614
F	-0.20154056	9.79467100	12.88834114
F	1.03562166	8.01130478	11.98660896
F	0.79042716	9.97683136	10.80860797
F	1.66219057	11.18700302	12.56531220
F	1.83457457	9.21956084	13.80344280
Au	6.12537693	4.44062872	8.40033994

P	6.47882985	5.30666060	6.26813020
P	6.15016497	9.56210976	8.23816037
N	7.75650047	7.47960086	7.27927110
N	8.71347763	8.70390687	8.97802007
C	7.90091833	6.48642674	6.23417401
H	7.94633556	6.98359437	5.24733312
H	8.82776499	5.90181958	6.35719864
C	7.11607812	8.72489872	6.89327702
H	7.85963394	9.45951986	6.53529204
H	6.41584346	8.50912098	6.07057013
C	8.63839213	7.51498035	8.34684273
C	9.41139034	6.40390468	8.74135682
H	9.28544003	5.42861348	8.27130587
C	10.38726484	6.60468561	9.70994119
H	11.03970784	5.77073370	9.98794325
C	10.52806744	7.85873219	10.32139495
H	11.29370471	8.04978867	11.07561755
C	9.63270107	8.85677120	9.94945493
H	9.65355135	9.84285907	10.42730604
C	5.07226086	6.25210238	5.61799845
C	3.92189144	6.41236128	6.40679579
H	3.89297267	5.99352796	7.41889000
C	2.82000209	7.10317782	5.89674689
H	1.92966218	7.23587347	6.51437367
C	2.86039717	7.63279269	4.60598648
H	1.99516823	8.17792906	4.22022052
C	4.00956661	7.49006736	3.82376186
H	4.05828238	7.92112438	2.82118035
C	5.11652172	6.80782458	4.32643911
H	6.01265837	6.72534606	3.70630458
C	6.86167642	4.05235056	5.02032126
C	7.46203024	4.39637161	3.79697180
H	7.78207037	5.42138396	3.58923858
C	7.66676573	3.41737936	2.82287266
H	8.14392938	3.68522291	1.87663942
C	7.27954175	2.09463606	3.06257620
H	7.43079681	1.33185858	2.29248961
C	6.71824592	1.73988480	4.29330203
H	6.44466873	0.69898691	4.48896468
C	6.51459111	2.71570852	5.27176523
H	6.10297740	2.44432266	6.25061258
C	4.51807109	9.87190802	7.49518102
C	4.36642537	10.36260713	6.18580598
H	5.22829704	10.49374199	5.52590695
C	3.09268994	10.66843058	5.70355250
H	2.97643166	11.06161773	4.68982697
C	1.96738722	10.47958056	6.51264595
H	0.97330753	10.72074302	6.12934798
C	2.10872981	9.97332093	7.80593435
H	1.23491972	9.83131219	8.44778574
C	3.38163086	9.66797683	8.29325505
H	3.50491811	9.28596594	9.31332555
C	6.94664870	11.18607358	8.38023423
C	7.27278045	11.71233944	9.63862276
H	7.00291795	11.14751299	10.53808497
C	7.94967732	12.93120666	9.73444872
H	8.20749983	13.34548153	10.71383364

C	8.30449704	13.62243207	8.57144715
H	8.85908412	14.56061550	8.63307941
C	7.95842110	13.11370262	7.31713201
H	8.23631551	13.66966901	6.41857896
C	7.27954741	11.89870056	7.21679465
H	7.04209434	11.50171675	6.22602461
P	10.70864250	3.34073763	6.41584727
F	9.18167997	3.54834896	6.98888773
F	12.22815516	3.15152517	5.82699275
F	10.99099293	4.93489137	6.72872492
F	11.23618742	2.96936478	7.90672592
F	10.36442403	1.75919314	6.15002168
F	10.19204004	3.72663531	4.91189107
